# Seasonal variations and shared latrine cleaning practices in the slums of Kampala city, Uganda

**DOI:** 10.1186/s12889-016-3036-7

**Published:** 2016-04-27

**Authors:** Japheth Kwiringira, Peter Atekyereza, Charles Niwagaba, Robert Kabumbuli, Charles Rwabukwali, Robinah Kulabako, Isabel Günther

**Affiliations:** Department of Sociology and Anthropology, School of Social Sciences, College of Humanities and Social Sciences, Makerere University, P.O. Box 7062, Kampala, Uganda; Department of Sociology, Kyambogo University, P. O. Box 1, Kyambogo Kampala, Uganda; Department of Civil and Environmental Engineering, Makerere University, P. O. Box 7062, Kampala, Uganda; Swiss Federal Institute of Technology –Zürich (ETH-Z) and Centre for Development and Cooperation (NADEL) Zürich, Zürich, Switzerland

**Keywords:** Seasons, Shared sanitation, Cleaning, Kampala, Slums

## Abstract

**Background:**

The effect of seasons on health outcomes is a reflection on the status of public health and the state of development in a given society. Evidence shows that in Sub-Saharan Africa, most infectious diseases flourish during the wet months of the year; while human activities in a context of constrained choices in life exacerbate the effects of seasons on human health. The paper argues that, the wet season and when human activities are at their peak, sanitation is most dire poor slum populations.

**Methods:**

A shared latrine cleaning observation was undertaken over a period of 6 months in the slums of Kampala city. Data was collected through facility observations, user group meetings, Focus group discussions and, key informant interviews. The photos of the observed sanitation facilities were taken and assessed for facility cleanliness or dirt. Shared latrine pictures, observations, Focus Group Discussion, community meetings and key informant interviews were analysed and subjected to an analysis over the wet, dry and human activity cycles before a facility was categorised as either ‘dirty’ or ‘clean’.

**Results:**

Human activity cycles also referred to as socio-economic seasons were, school days, holidays, weekends and market days. These have been called ‘impure’ seasons, while the ‘pure’ seasons were the wet and dry months: improved and unimproved facilities were negatively affected by the wet seasons and the peak seasons of human activity. Wet seasons were associated with, mud and stagnant water, flooding pits and a repugnant smell from the latrine cubicle which made cleaning difficult. During the dry season, latrines became relatively cleaner than during the wet season. The presence of many child(ren) users during school days as well as the influx of market goers for the roadside weekly markets compromised the cleaning outcomes for these shared sanitation facilities.

**Conclusion:**

Shared latrine cleaning in slums is impacted by seasonal variations related to weather conditions and human activity. The wet seasons made the already bad sanitation situation worse. The seasonal fluctuations in the state of shared slum sanitation relate to a wider malaise in the population and an implied capacity deficit among urban authorities. Poor sanitation in slums is part of a broader urban mismanagement conundrum pointing towards the urgent need for multiple interventions aimed at improving the general urban living conditions well beyond sanitation.

## Background

Studies have shown that morbidity and mortality are at times season mediated. This is because seasons affect human activities and service delivery especially among the poor [1, 2]. Sanitation related functions of solid waste collection and management also have seasonal variations. Mechanisms that permit human populations to better mediate the relationships between themselves and climatic -environmental conditions in which disease organisms are more controlled tend to come with progress that insulates populations against seasonal shocks [3, 4]. When health outcomes least vary with season, then the (annual) distribution of morbidity (the rate of disease, illness or sickness prevalence and incidence in a population) and mortality (the incidence of the number of deaths in a population) does not display much overall variation from 1 month to another, and from one season to another [5, 6]. In Uganda, most primary healthcare diseases are related to the season of the year. For instance, the two Malaria transmission seasons; (August to October) while the second transmission season is linked to the two wet seasons that the country experiences (from April to September) [7–9]. Previous cholera episodes in Kampala city have also occurred during the wet season implying poor and inadequate sanitation practices [10–12].

Kampala city presents a semblance of wellbeing for everyone. However, this is false, given the evidence in slum sanitation and the makeshift living conditions therein that are adversely affected by seasonal changes [13, 14]. Flooding in urban areas is not just related to heavy rainfall and extreme climatic events; it is also related to changes in the built-up areas themselves [15].

Human excreta management is a fundamental aspect to public health in slums since most of the pathogens are of faecal origin [16, 17]. Pathogens form a major cause of disease transmission due to their presence in human excreta and when mixed with wastewater, the pathogens flow downstream and spread in the environment especially during flooding [18, 19]. Flooding is a major problem in all informal settlements in the developing world [15, 20]. A recent study on the performance of pit latrines in the slums of Kampala shows that the level of pit content was predicted by rain or storm water which, also affected the functioning of these facilities depending on whether or not they were flooded [21].

To date, no known study has evaluated the effects of seasons on slum shared latrine access and use, let alone cleaning in the slums of Kampala. The paper aims at presenting the influence of seasonal variations on the cleaning practices among shared latrine users in the slums of Kampala.

## Methods

We assessed shared sanitation cleanliness in six zones shown in Table 1 in Kampala city for a period of 6 months (from December 2014 to May 2015).

**Table 1:** Selected slum zones and their location in Kampala City

City division	Parish	Zone name
Kawempe	Makerere –III	Dobbi
Central	Kamwokya –II	Kisenyi −1
Makindye	Nsambya	Gogonya
Makindye	Kabalagala	White Nile
Makindye	Kabalagala	Kisaasizi
Makindye	Kibuye –I	Jjuko

Once a fortnight, a photograph of each of the observed sanitation facilities was randomly taken and dully logged on the monitoring sheet for each zone. Data was also collected from 18 Focus Group Discussions with three FGDs per zone. Each FGD was homogenously composed of 8–10 participants. FGD participants were adult female or male residents in the study zone for more than 5 years. Ten Key Informant Interviews (with landlords, local leaders and agencies that provided sanitation to slum dwellers) were conducted. Key informants were; persons involved in sanitation for the urban poor, property owners in study areas, public health providers (directly or indirectly) and leaders (technical or political) in the city. Observation for feacal contamination and other forms of dirt such as urine and other waste and dirt was made weekly by research assistants using a checklist so as to obtain the state of cleanliness for each sanitation facility. On the basis of this, the cleanliness of each shared sanitation facility was evaluated and recorded and feedback given to the shared facility users.

### Ethical issues

The study protocol was approved by the Research Committee in the School of Social Sciences, College of Humanities and Social Sciences Makerere University. This committee considered all technical and ethical issues of the study. Clearance was also obtained from the local leaders in the respective slum zones. An introductory letter issued by Makerere University was presented to local leaders in addition to explaining the purpose of the study, confidentiality, voluntary participation; anonymity and freedom to withdraw from the study were clearly explained [22, 23]. Verbal consent to participate in the study was obtained from all study participants. Participants were free to withdraw from the study if they felt uncomfortable. No persons lacking capacity to consent were enrolled or involved in the study. In addition, study participants’ identifiers are not presented. The need for confidentiality was emphasized during training of research assistants prior to conducting of the study [22]. With the study findings being published, this shall minimize community research fatigue and wastage of valuable resources [24].

### Quality assurance

Four graduate research assistants who knew most of the local languages (Luganda, English, Swahili, Lusoga and Runyakitara) in the slums of Kampala were recruited. In addition, these research assistants had experience in conducting focus group discussions and interviews. Research assistants were trained for two days and also participated in pre-testing the data collection tools for one day. The pre-testing zone (Butaka-Bukirwa) did not take part in the main study. After pre-testing the tools, adjustments were made and data collection commenced. Daily field review meetings were held to capture emerging issues for follow up and to provide guidance for further data collection.

### Data management and analysis

After fieldwork, toilet cleaning data were then entered in excel. The trend of cleanliness for shared facilities was then subjected to analysis taking into consideration the different seasons namely; wet, dry, (also called ‘pure’ seasons) and human activity seasons (referred to as ‘impure seasons’) namely market and school days. Cleaning data were analysed for seasonal variability before the final cleaning status of ‘clean’ or ‘dirty’ was determined basing on the photos taken weekly, field visits and reports from cleaning logs and the fortnight user meetings.

Qualitative data analysis followed a content thematic approach advanced by Graneheim and Lundman [25] to identify both manifest and latent content. Raw data from FGDs and interview scripts were independently read several times to identify emerging themes and sub-themes. Joint discussions with research assistants were held to compare themes and sub-themes identified; a process that led to development of a unified list of codes for use in data analysis. The identified themes and sub-themes were used to code data. Sub-group analysis was done, which involved examining the themes and sub-themes. The data coding process began during collection and went on until the end. This enhanced continuous analysis while serving as an analytic method for coding and analysis [26, 27]. Raw data consisting of interview transcripts, participant observation, field notes and photographs were coded. In first cycle coding, data were bigger in magnitude with the coding outcome ranging from a single word to a full sentence and sometimes to a set of sentences covering an entire page. The coding process proved heuristic and served as an exploratory technique to the seasonal variations in shared latrine cleaning.

‘Dirty’ facilities were those having paper or other used material containing feaces or urine on the floor, having flies or other vermin such as cockroaches; showing evidence of mud on the floor or walls. Such facilities posed a danger of contaminating the user with human waste. ‘Clean’ facilities were those that one could use without getting in contact with human excreta (Table 2).

**Table 2:** Key to coding photos

Category	Visual basis of categorization
Dirty facilities were those with feaces, urine, flies or mud on the floor or walls. Such a facility would not be used without getting in contact with human excreta and at great discomfort and compromise to personal aesthetics. Such facilities were mostly shunned for open defecation	
Clean facilities were those with no feaces, urine, flies or mud on the floor or walls. Such a facility could be easily used without fear of getting in contact with feaces.	

## Results

**Fig. 1:**
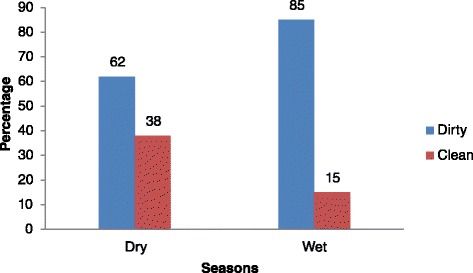
Shared latrine cleanliness and seasons in Kampala slums (*N* = 50)

Findings show that shared sanitation facilities became dirtier during the wet season and became cleaner during the dry season. However, due to climate change, these wet and dry seasons have been changing and continue to change, with wet seasons becoming more wet and the dry seasons becoming drier, with this pattern being quite unpredictable. The key issue in this paper were the effects of these seasons on shared latrine cleanliness for the slum dwellers. Figure 1 shows that the status of shared sanitation facilities varied by season (wet or dry), with more facilities getting dirtier during the wet season.

**Fig. 2:**
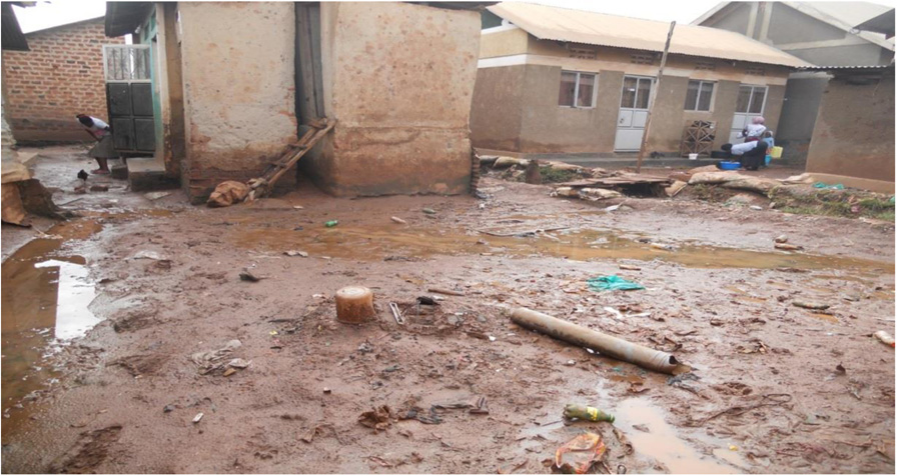
A muddy yard that complicates latrine use and cleaning

Findings in Fig. 1 show that, there were more (85 %) dirty latrines during the wet season, than during the dry season (62 %). The proportion of clean shared facilities increased during the dry seasons to 38 % from 15 % in the wet season. The wet season made the already poor environmental conditions worse especially the aesthetics of non-lined pits and poorly constructed facilities that usually flooded making their use impossible and harmful. During the wet season, improved sanitation facilities polluted less than the unimproved facilities. Generally, during the dry season, both improved and unimproved facilities exhibited better sanitation scores than during the wet season. In the dry season, pits did not flood, were with less stagnant water in the adjacent yards (Fig. 2), there was less foul smell in the latrine cubicles and less manual emptying of pits. A combination of these factors made the floors easer to clean however, during the dry season, users complained of water scarcity. Studies on slum water access and supply have linked seasonal water scarcity to low supply capacity of service providers leading to water rationing as well as failure to pay for the supplied water leading to disconnections even where connections exist [28–32].

Unimproved shared sanitation facilities were more prone to seasonal disuse, failure and abuse than the shared improved facilities. The one main effect of wet seasons was the discounting of improved sanitation benefits on account of wider poor environmental health. Although improved latrines are designed to be more resilient to seasonal variation and other types of seasonal shocks [20, 21, 33]; the slum conditions greatly undermined this position. To the majority of the slum dwellers in the long-run, lined pits tend to have high capital expenditure (CAPEX) and lower operational expenditure (OPEX), and therefore if well used and maintained became cheaper and a more viable excreta disposal technology especially in slum conditions [34]. Table 3 presents a summary of the comparison between the improved and unimproved sanitation facilities during the wet season as well as the seasonal factors behind the status of each facility typology.

**Table 3:** Shared latrine category, cleanliness/dirtiness in the wet season

Reasons for latrine status (clean or dirty)		
	Reasons for being clean	Reasons for being dirty
Improved	• Had raised and lined pits. Pit contents not affected by high water table or flooding (surface or underground) • Due to frequent down pours, (those 1that could harvest rain 4water) there was water to clean	• Some facilities were inaccessible due to flooding in the yard. • Floors were challenging to keep clean due to muddy (unpaved) yards; the mud was carried underneath users’ feet and left on the latrine floor. • There was flooding of the latrine pit and the floor by surface run-off. • The dirty slum environment did not encourage keeping the latrines clean • In low lying areas, some houses were abandoned and this disorganized the latrine cleaning arrangements thereby accounting for setbacks in the cleaning of these facilities. • People perceived venturing out to access latrines more dangerous when it was raining at night. This forms some involuntary choices that lead to poor latrine use. • Some adults resorted to squatting near the entrance/door, around the facility or used flying toilets.
Unimproved	• The clean unimproved facilities were very few and were mainly located in low water table and relatively well drained areas in the slums of Gogonya, Jjuko and Kisaasizi	• Floors were challenging to keep clean on account of muddy • (unpaved) environments. • Pit flooding from both underground and surface sources made latrine use, access and cleaning complex and almost impossible. • Rain over a long period of time made adults fear that the slab would collapse due to the weakened soils and the poor construction standards. • During the wet season, after the collapse of some structures, there was increased free riding and open defecation (OD) • Pits smell and made users uncomfortable • There was flooding of the latrine pit and the floor by surface run-off water that also carried pollutants. • The generally dirty slum environment did not encourage keeping the latrines clean • People perceived venturing out to access latrines more dangerous when it was raining at night. This forms some involuntary choices that lead to poor latrine use. • Some adults resorted to squatting near the entrance/door, around the facility or used flying toilets. • Some people took advantage of the stagnant water to empty latrines in the environment and also to open defecate • In low lying areas, some houses were abandoned and this disorganized the latrine cleaning arrangements thereby accounting for setbacks in the cleaning of these facilities.

The few users that had the means of harvesting rain water (having a gutter and a water storage container) had better access to water for cleaning during the wet season although other challenges such as muddy yards (Fig. 2) remained.

Sanitation facilities such as the one shown in photo one usually had a foul smell which constrained access and use.

**Fig. 3:**
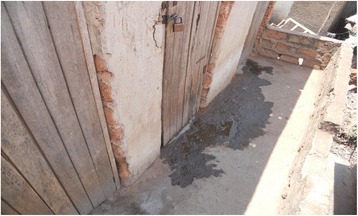
Effects of flooding on a latrine floor

Some of the background challenges to cleaning shared sanitation facilities during the wet season related to access and use due to flooding yards which made proper use unpleasant and sometimes impossible. As a result, latrine floors become dirtier during the rainy season than the dry season. Latrines located in high water table areas sometimes filled and flooded resulting in spillage of human waste on the floor (Fig. 3) and in the wider environment.

**Fig. 4:**
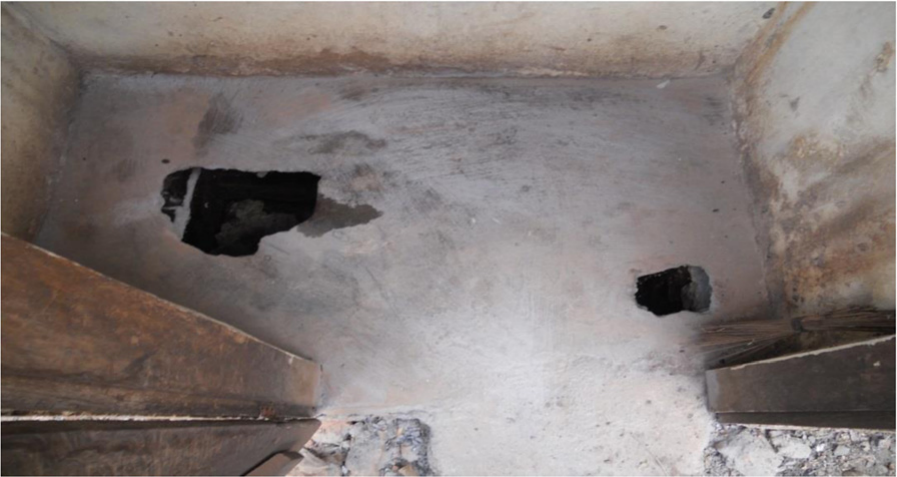
A weak slab over a pit full of water with a foul smell

The latrine floor shown in Fig. 3 makes it unattractive to enter, use and clean. The user cleaning intentions are curtailed by the filthy floor. In many cases, such latrine facilities had weak floors that were a threat to personal safety (Fig. 4). One FGD participants narrated thus;

*‘When it rains, the whole area becomes like a lake. It becomes hard to access the latrine since it is near a drainage channel. Even after you get there, the floor is all flooded with feaces from the pit.’* FGD women, Kisaasizi

**Fig. 5:**
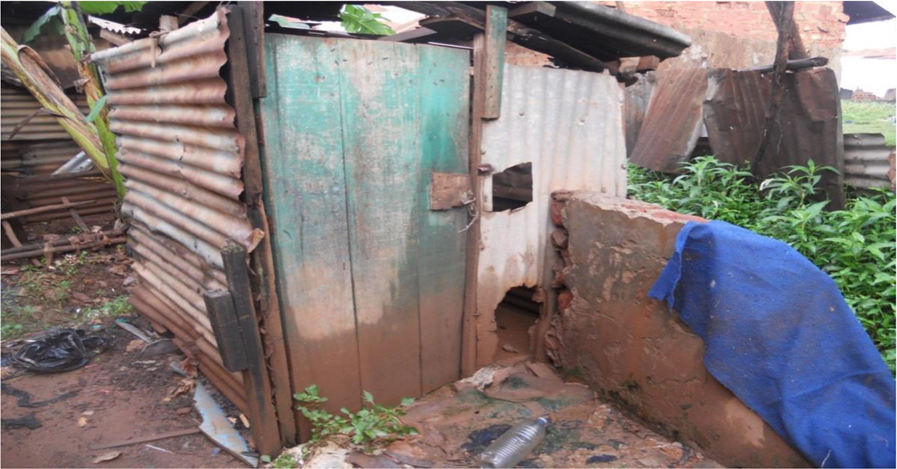
A degraded and abandoned latrine due to flooding

Flooding was also found to affect the poorly located facilities on account of storm water and groundwater infiltration (Fig. 5).

An FGD participant interjected that;

*‘Even if the latrine was well used; the amount of dirt on the floor confuses me when I want to clean, am not sure, whether the dirt is feaces or mud brought by people’s feet. The leaking roof also makes the floor look as if it is covered in urine. That’s why many people are unwilling to clean during the wet season.’* FGD women, Kisaasizi

One woman in Dobbi zone related the wet season to a smelling latrine and the motivation to use a flying toilet;

*“When it starts raining, you can only use that latrine when you are sure that you must take a shower; if you do not shower, no one can stand you… Every time you leave the latrine, the people you meet can tell where you have been. That is why I prefer using the polythene bag at home.”*

The problem of smell was more pronounced during the wet (rainy) season. Overall, irrespective of the facility type, the rainy season presented a number of challenges. Most cleaning dropouts were also noted during the wet seasons. The rainy seasons were associated with flooding especially in Dobbi zone. As a coping mechanism, some tenants relocated to places in and out of the zone that were not flooding; in such cases, the cleaning arrangements were disrupted which resulted in more dirty sanitation facilities during the wet season than the dry season(s).

### Impure seasons

**Fig. 6:**
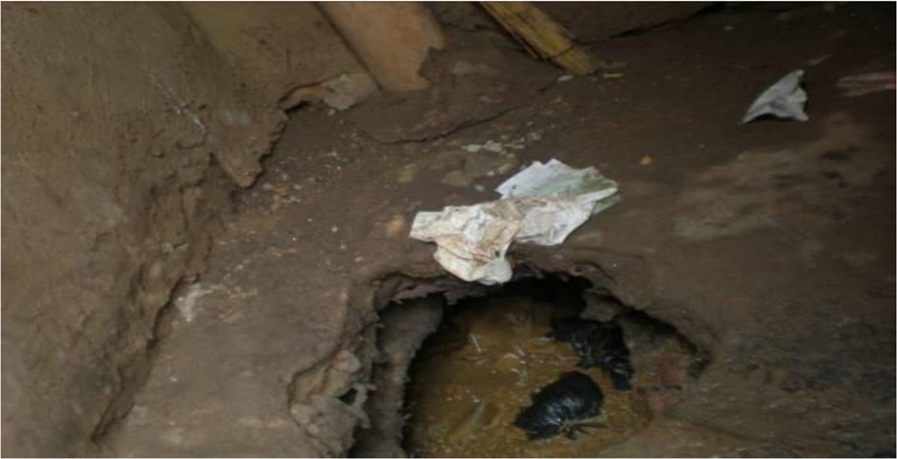
Unusable pit latrine that was not child friendly

Data for impure seasons also referred to as sub-seasons was obtained during market days, week-ends, public holidays, school days and (school) holidays. During these ‘seasons’ sanitation status rotated around human activities that influenced shared cleaning. Households with school going children as well as those sharing with such households, weekends and holidays proved a challenge to shared access and cleaning. It was reported that children misused the latrines and did not properly clean them. This was especially mentioned where children were less than 10 years. There was a specific compliant about mothers who sent their children to empty potties in latrines. Such children were reported to empty the potty on the floor instead of emptying it in the pit probably due to fear of getting too close to the sometimes big squat holes (Fig. 6).

Even then, when children were encouraged to use the latrine instead of the potty, they usually left the hole and floor soiled which left their parent arguing over ‘*whose child*’ was to blame. For the lockable shared facilities, many child users led to the frequent loss of keys, which left such a facility accessible to the wider public. This unrestricted access to a shared facility caused another array of cleaning problems. Such practices and occurrences affected shared cleaning during school and non-school time for households sharing sanitation with schools and households with school going children respectively. To households, with school going children, school days were the best time for shared latrine cleaning. This was on account of few and more responsible users. School days saw few children at home, in which case, the home facilities became cleaner as the school ones got dirtier for households that shared these facilities with schools (February-April, May-August, and September-November). Irrespective of the sanitation user category, all shared sanitation facilities became dirtier during the wet season. (School holidays occur during the months of January, April, August and December). Therefore, the school calendar had implications for shared sanitation facility cleanliness. This was the reverse for households sharing sanitation with schools; school days were sanitation nightmares. Table 4 summarizes the school time and holiday time shared sanitation cleaning challenges.

**Table 4:** School Cycle and shared cleaning in Slums

School time	Week-ends and holidays
• Many users and improper use/misuse. • At any one time, some of the children have diarrheal diseases, under such conditions, they soil the latrines and they do not clean them due to lack of cleaning materials. • Majority of the children do not clean toilets after use. • Households sharing facilities with schools faced cleaning and emptying challenges during school days. • For households with many school going children, their facilities were cleaner when children were at school. • Quick pit filling discouraged frequent and proper latrine use	• Latrines are relatively cleaner with more adult(s) than child/ren users. • Day school facilities were cleaner on weekends and (public) holidays than school days. • Frequent use by young children made it difficult to keep clean shared facilities with children at home the whole day. • For households with school going children, locking was problematic as keys and padlocks were easily lost by children. This led to unrestricted use by the wider community.

For households and communities that shared sanitation facilities with schools, weekends and school holidays were the best for cleaning on account of less usage, few(er) users and fewer break-ins. When schools were open, the shared facilities were not well used, poorly cleaned and not well maintained. This challenge was most pronounced for water borne and ecological sanitation facilities that many children were not able to use properly.

For the few sanitation facility users that had the means of harvesting rain water, the rainy season provided water for cleaning. However, other challenges such as muddy yards (Photo 1) which made the latrine floors dirty remained.

### Market days

Among the study zones, only one zone had a weekly road side market. The sanitation activities in such a market facility had negative effects on neighbouring communities. Local leaders noted that, the sanitation challenges were worse during peak shopping seasons of children going back to school and the festive seasons (Easter and Christmas) when shoppers are so many. The fact that this market was once a week and cheap, made it a favourite city wide destination for low income shoppers. The other zones with gazetted and daily markets did not experience sanitation ‘shocks’ as did the zone with the unplanned market. The market day was also known a ‘pollution day’.

‘*The market here is every Friday. We have tried to address the issue of sanitation but we are failing because the people are just so many. The public latrine is overwhelmed by the number of users. Because of this, people resort to squatting in every corner. If you want to see the dirtiest latrines come here on Fridays…since the market goes on till late in the night; when darkness falls, everywhere becomes a latrine since it is a matter of squatting*.’ Local leader, Kisenyi 1, Zone, Kamwokya Parish

It was clear that, the weekly open air market was not organised to meet the sanitation needs of the many buyers and sellers. This was worsened by the general sub-optimal sanitation infrastructure in all the slums of Kampala.

Poorlyconstructed sanitation facilities that allowed storm or rain water entry compromised facility hygiene, function and cleaning efforts. The rainy season was detrimental to the extent that the sanitation facility had a leaking roof or none at all. Other facilities did not have a water tight system (for waterborne facilities) or containing chamber, this means that, during the rainy seasons there is more pollution caused by discharge from latrine pits and manholes in the case of water borne facilities. Generally, facilities in flood prone areas became less functional during the wet season. On the whole, the rainy season and other peak use seasons made operation and maintenance difficult which were background factors in covert open defecation including use of flying toilets, poor cleaning and subsequent facility abandoning [13, 14].

Most yards were usually flooded during the rainy season, as such, some households preferred to pour all the wastewater in the latrine pit to minimise on flooding. This increase in the liquid volume in the pit contributed to pit filing and flooding [21]. The dirt in the latrines during the wet season was associated with an increased presence of flies (due to bad smell) which further discouraged cleaning of these facilities. During the rainy and high use peak seasons, the almost full and not well constructed latrines respectively exhibited worse cleaning indices.

## Discussion

Due to the varying nature of challenges facing shared sanitation access, use and cleaning (women, children, the sick and elderly) in Kampala slums, there were equally different coping mechanisms adopted with equally changing outcomes on the environment with most outcomes being poor sanitation practices at different levels [14]. During the rainy season, the environment widely had a stench of human waste (urine and feaces). This was linked to the fact that during the rainy season unscrupulous residents opened the pit containment chambers of latrines so that the feacal contents could flow into the drains downstream with the storm water [15, 17, 35]. It is evident that slums exhibit unique characterisations that make them neither rural, nor urban, by presenting a unique blend of concentrated disadvantages and deprivation for the poor slum dwellers, which 331 makes them (slum dwellers) vulnerable to seasonal variations. Generally in all the study zones, dirty facilities discouraged further use, while concomitantly encouraging abuse and complete shunning of the available dirty facilities, in favour of open defecation.

The variation between dirty and clean sanitation facilities for slum dwellers is extreme and more pronounced during the rainy season. Paradoxically, the sanitation related advantages associated with improved facilities were greatly constrained and sometimes diminished during the wet season on account of terrain and poor environmental health resulting from flooding and poor drainage [10, 11, 35–37]. These factors also affected the sanitation and hygiene indices across the slums which explains the previous occurrence of waterborne and sanitation related ailments including cholera in Kampala slums [10, 35].

Latrines in slums tend to flood or fill up during the rainy season, with the general use of sanitation facilities going down on account of the wider contamination in the neighbourhood [38, 39]. While there is plenty of water in the slum environment this water may be unusable and in some cases does not serve a good sanitation purpose. The sanitation challenges during the rainy season relate to access, safety, convenience and ultimately cleaning of the available sanitation facilities [39]. On the whole, rainy seasons made the sanitation situation direr for the slum population. Therefore, latrine access, use and cleanliness is much more complex during the rainy season than any other time of the year depending on; type of facility (improved or unimproved), the structural integrity of the facility user type numbers as well as location. Some sanitation facilities in slums are located in the most unusable of places such as near or over drainage channels [40].

There is a need to revisit the methods and strategies for school sanitation in slum areas. This is because factors related to water, sanitation and hygiene affect children’s right to education in many ways [41–43]. In an atmosphere of poor health, children are unable to realise and fulfil their education potential. For example, every year, 400 million school going children are infected by intestinal worms, which, research shows saps their learning abilities [43]. School sanitation should be linked to household and community capacity especially empowering parents and guardians to encourage and practice good sanitation behaviour along with their school going children.

The wider decline in shared sanitation facility cleanliness was encouraged by the broader malaise in the general slum conditions. Since slum conditions were dirty, there was no motivation to only keep the sanitation facilities clean. The most common unhygienic human waste disposal practice during the wet season was the indiscriminate disposal of feaces on the open dump sites. This practice was further encouraged by the poor garbage collection during the wet seasons due to absenteeism of garbage collectors in addition to poor (vehicular) access due to flooding as well as the convenience of dumping garbage and human waste in stagnant 365 water and drainage channels [44, 45].

Wet and rainy seasons worsen many other causes of poor sanitation including poor access to latrines (especially at night for females), many users, the ease of (human) waste disposal in the environment, quick pit filling and carelessness among users, and the lack of cleaning materials. That this is the case, is in line with findings from previous studies on sanitation in Kampala’s slums [13, 14]. Seasonal setbacks seriously reduced the 376 benefits of improved sanitation facilities to almost the level of performance only comparable to the unimproved sanitation facilities with their attendant sanitation challenges especially environmental pollution. Different seasons have an effect on slum sanitation and this impact is mediated by human and non-human factors such as age (children and the elderly) and gender (women and girls) [13, 14], as well as facility type and location especially solid waste management, wastewater management, water supply, topography and the water table [40, 46]. Therefore, improved sanitation practices and interventions are more likely to enhance cleanliness of improved facilities which are also better located than those facilities that are poorly located. The sanitation challenge in slums points to institutional weaknesses and household poverty; patronage and the lack of settlement planning and the poor enforcement of housing regulations that partly lead to the growth of slums in the first place. Almost by default, slums have few improved sanitation facilities [11, 37].

During the wet season, sanitation efforts and strategies need to be scaled up. This should be in addition to other community wide interventions that address seasonal challenges affecting slum sanitation in different ways. Some of the issues include, need to control flooding and improved drainage, improve water supply and access as well as improved garbage management and drainage so as to improve the living conditions of slum dwellers [13, 47]. Therefore, sanitation is not a stand-alone component to slum life.

The study was limited by a small sample size. The small sample of willing participants to take on the responsibility of improving shared sanitation in slums could also be indicative of the fact that sanitation may not be among the top priorities for the slum dwellers. While improved sanitation awareness is good and has its place; improved livelihoods, planning and welfare have their practical contribution. The results of this study are relevant in informing policy makers and technical staff on the role of seasonal variations in sanitation status among the urban poor. Weather proof sanitation facilities were relatively cleaner than those that had no roof and not lined; occasioning other associated malfunctioning such as water infiltration into the pit that got worse during the rainy season. Weather proof facilities have multiple positive health benefits for women and children who are the most deserving user categories.

## Conclusion

The study shows that, we cannot de-link sanitation from other aspects of urban poor areas especially the quality of the population, the capacity of urban authorities to deliver services including urban governance, urban planning and enforcement and waste management [46]. Therefore, seasonal sanitation variations and the attendant challenges are part of a wider urban management and governance conundrum. This reality points towards the need for various interventions; both short term and long term, aimed at improving the urban living conditions well beyond sanitation. Poor slum sanitation has many causes and the discussion of seasonality should not distract attention from those which are structural, technical, geo-political, environmental and socio-cultural. The issue of slum sanitation needs to be revisited in view of the multiplicity of contextual challenges than a mere eagerness to replicate or implement previous blue prints from other urban sites around the globe. This season-sanitation inquiry can be further studied based on these findings especially by widening the scope of this analysis. Sanitation is a complex problem that requires multi-level interdisciplinary actions for resolving this complexity. Kneejerk reflexes and actions are inconsequential in the long run.

### Ethics approval and consent to participate

The study was approved by the Research Committee in the School of Social Sciences, College of Humanities and Social Sciences Makerere University, with verbal consent to participate in the study being obtained from all study participants.

### Consent for publication

Not Applicable.

### Availability of data and materials

Additional supporting file Submitted with manuscript.

## Abbreviations

CAPEX: capital expenditure
ETH-Z: Swiss Federal Institute of Technology –Zürich
FGD: focus group discussions
KCCA: Kampala Capital City Authority
KII: key informant/ interview
NADEL: centre for development and cooperation Zürich
OPEX: operational expenditure
SSA: Sub Saharan Africa
WHO: World Health Organisation
